# Identification of a novel lytic peptide for the treatment of solid tumours

**DOI:** 10.18632/genesandcancer.18

**Published:** 2014-05

**Authors:** Claudia Szczepanski, Olav Tenstad, Anne Baumann, Aurora Martinez, Reidar Myklebust, Rolf Bjerkvig, Lars Prestegarden

**Affiliations:** ^1^ NorLux Neuro-Oncology, Dept. of Biomedicine, University of Bergen, Norway; ^2^ Centre de Recherche Public de la Santé, Luxembourg, Luxemburg; ^3^ Cardiovascular Research Group, Dept. of Biomedicine, University of Bergen, Norway; ^4^ Biorecognition Group, Dept. of Biomedicine, University of Bergen, Norway; ^5^ Dept. of Dermatology, Haukeland University Hospital, Bergen, Norway

**Keywords:** Antimicrobal lytic peptides, cationic lytic peptides, cancer treatment, membrane disruption

## Abstract

Originally known as host defence peptides for their substantial bacteriotoxic effects, many cationic antimicrobial peptides also exhibit a potent cytotoxic activity against cancer cells. Their mode of action is characterized mostly by electrostatic interactions with the plasma membrane, leading to membrane disruption and rapid necrotic cell death.

In this work, we have designed a novel cationic peptide of 27 amino acids (Cypep-1), which shows efficacy against a number of cancer cell types, both *in vitro* and *in vivo*, while normal human fibroblasts were significantly less affected. Surface plasmon resonance experiments as well as liposome leakage assays monitored by fluorescence spectroscopy revealed a substantial binding affinity of Cypep-1 to negatively charged liposomes and induced significant leakage of liposome content after exposure to the peptide. The observed membranolytic effect of Cypep-1 was confirmed by scanning electron microscopy (SEM) as well as by time-lapse confocal microscopy. Pharmacokinetic profiling of Cypep-1 in rats showed a short plasma half-life after i.v. injection, followed mainly by retention in the liver, spleen and kidneys. Extremely low concentrations within the organs of the central nervous system indicated that Cypep-1 did not pass the blood-brain-barrier.

Local treatment of 4T1 murine mammary carcinoma allografts by means of a single local bolus injection of Cypep-1 led to a significant reduction of tumour growth in the following weeks and prolonged survival. Detailed histological analysis of the treated tumours revealed large areas of necrosis.

In sum, our findings show that the novel cationic peptide Cypep-1 displays a strong cytolytic activity against cancer cells both *in vitro* and *in vivo* and thus holds a substantial therapeutic potential.

## INTRODUCTION

Naturally occurring antimicrobial peptides (AMPs) represent a first-line defence of eukaryotic cells against bacteria, protozoa, fungi and viruses [1-3]. Originally known as host defence peptides [4] or defensines [5], many AMPs, as well as their synthetic variants, have been found to exert a broad spectrum of antimicrobial and anticancer effects, while often affecting normal mammalian cells to a far lesser extent [6-14].

These cytolytic effects of AMPs are largely attributed to their cationic nature [15, 16], leading to electrostatic interactions with negatively charged cell membranes [17]. AMPs may either cause plasma membrane destabilization or cell lysis, which leads to rapid necrotic cell death. Alternatively, some AMPs permeate the cell membrane and then destabilize or disrupt the target cell's mitochondrial membranes instead, resulting in a pro-apoptic effect [18-20]. In addition, a few AMPs have been found to inhibit angiogenesis in addition [11, 13, 20, 21]. Since their lytic potential is neither receptor-mediated nor dependent on the cellular replication cycle, AMPs targets multi-drug-resistant cancer cells successfully and thus represent a promising group of novel oncolytic treatment agents [12, 19, 22-25].

Normal mammalian cells are known for their asymmetric distribution of electrostatically neutral zwitterionic phospholipids within their membrane bi-layer [26]. Their outer membrane leaflets consist mainly of cholinophospholipids with neutral polar head-groups, while the aminophospholipids with negatively charged headgroups, such as phosphatidylethanolamine (PE) and phosphatidylserine (PS) are mainly found in their inner membrane leaflets [27]. This asymmetrical lipid arrangement is thought to promote mechanical membrane stability, whereby especially interactions between the PS on the inner leaflet and skeletal proteins seem to entail membrane modulatory effects [28].

In contrast, neoplastic cells often overexpress PS in the outer leaflet of their cell membranes [12, 16, 25, 29-34], resulting in a slightly more negatively charged membrane than normal cells. This promotes the interaction with AMPs and may therefore be partly responsible for the relative tumour cell selectivity [11]. Additional membrane surface characteristics of malignant cells, such as a lowered cholesterol content [36-38], alterations in the glycolysation of membrane-associated glycoproteins and glycolipids, [39-42] and the expression of certain surface proteoglycans [14, 43-46] as well as the presence of filopodia and microvilli [47] may, in addition, enhance the susceptibility of tumour cells towards AMPs.

In this work, we designed a library of 96 peptides with potential anti-tumour activity *in silico*. These peptides were originally derived from active sites of known tumour suppressors and oncogenes, as part of an attempt to identify specific peptides that might restore the function of inactivated tumour suppressors or interfere with oncogenes by competitive inhibition. The resulting molecules were synthesized and screened on monolayer cultures of the U87 human glioma cell line. While three out of the 96 peptides displayed anti-tumoural properties *in vitro*, we decided to focus on the most potent peptide. This peptide was originally derived from the tumour suppressor Axin2 (aa 126-140), which is a central protein in the Beta-Catenin pathway. However, instead of mimicking the functions of Axin2, we found it to have a lytic effect on the plasma membrane of cancer cells.

To avoid proteolytic degradation *in vivo*, we substituted the naturally occurring L-amino acids of the original *in silico* formula with their D-amino acid enantiomeres, as these are not recognized by serum proteases [48-50]. This resulted in a stable cationic peptide of 27 D-amino acids, designated Cypep-1, with a theoretical isoelectric point (pI) of 11.81 and a molecular weight of 3492.16 U [51].

We then proceeded to evaluate the efficacy of Cypep-1 on a total of 17 cell lines *in vitro* (4 osteosarcoma, 6 glioma and 3 mammary carcinoma) as well as 3 normal human cell lines (2 fibroblast cell lines and normal human osteoblasts). In addition, we assessed the cytotoxic effect of the peptide on 4T1 murine mammary carcinoma cells *in vitro*, as well as on corresponding allografts *in vivo*. The observed membranolytic effect of Cypep-1 was confirmed by scanning electron microscopy (SEM) as well as time-lapse confocal microscopy. Structural and mechanistic insights on the interaction of Cypep-1 with biological model membranes were obtained by surface plasmon resonance (SPR) and liposome-content leakage assays monitored by fluorescence spectroscopy. Furthermore, the pharmacokinetic profile of Cypep-1, its *in vivo* toxicity and mode of action were delineated.

## RESULTS

### Peptide Design and Screening

The initial screening of the peptide library on U87 glioma cells showed that three out of the 96 peptides designed *in silico* had a significant cytotoxic effect. The most potent peptide was selected for further development (Suppl. Fig.1, upper panel).

We then compared the anti-tumour efficacy of the original peptide formula (all L-amino acids) to the two sub-variants: D-aa (ri) and D-aa by testing them in various concentrations on U87 cells. Crystal violet staining showed that, while all three peptide formulations had a cytotoxic effect, the formulations made of D-amino acids proved to be more potent than the parent L- amino acid formula (Suppl. Fig.1, middle panel). The superior anti-tumour activity of the D-aa variant was verified by light microscopy. This showed that the monolayer of a U87 cell line had contracted considerably after 3 hours exposure and extensive cell loss was observed (Suppl. Fig.1, lower panel).

### Scanning Electron Microscopy (SEM)

**Figure 1:**
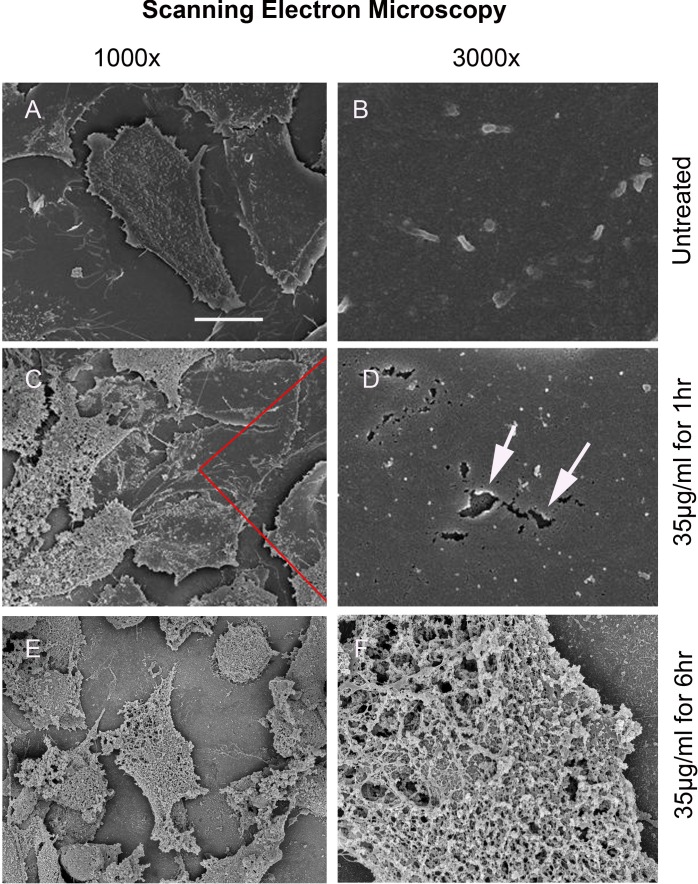
Scanning electron micrograph (SEM) of the mammary carcinoma cell line 4T1 demonstrates disintegration of the plasma cell membrane after treatment with Cypep-1 A-B) Untreated cells. C-D) After 1 hour incubation E-F) After 6 hours incubation.

At a magnification of 1000x, the scanning electron micrographs of untreated 4T1 mammary carcinoma cells showed cells with a flattened morphology, featuring membrane ruffles and filopodia (Fig. 1A). A more detailed micrograph at 3000x revealed small microvilli protruding from an intact cell membrane (Fig. 1B).

The first signs of cell membrane destruction became apparent after 60 minutes incubation with 35 µg Cypep-1/ml, (Fig. 1C), whereby numerous holes in the plasma membrane appeared (arrows in Fig. 1D). SEM micrographs taken after 6 hours exposure revealed a total disintegration of the plasma membrane (Fig. 1E and F).

### Cell Viability Assays

### MTT Viability Assays

**Figure 2:**
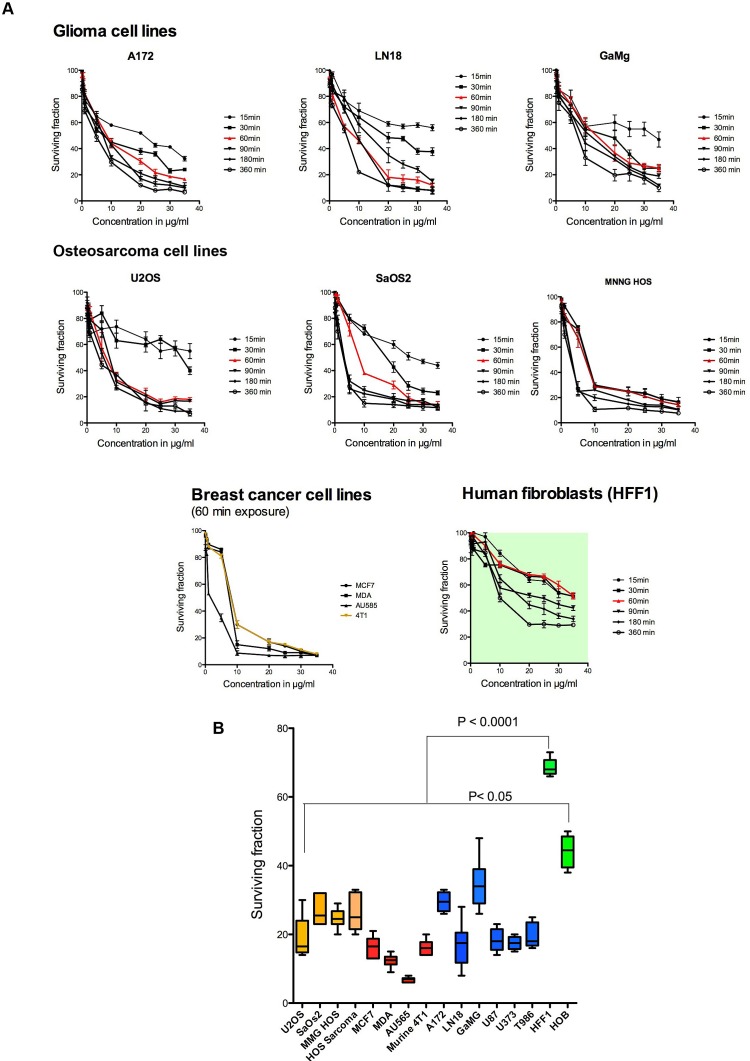
A) Dose and time dependent effect of Cypep-1 on different cancer cell lines and normal fibroblasts. B) Surviving fraction of the osteosarcoma (orange), breast cancer (red) and glioma cell lines (blue) after 60min. exposure to 20µg/ml Cypep-1. Green bars represent two normal cell lines HFF1 and HOB.

The cytotoxic effects *in vitro* of Cypep-1 clearly showed a dose and time dependent response for all cell lines (Fig. 2). As seen in Fig. 2B (lower panel), the average surviving fractions after 60 min of incubation with 20 µg Cypep-1/ml varied between 7% (mammary carcinoma AU 565; most sensitive cell line) and 33% (glioblastoma GaMg; least sensitive cell line). The two normal cell lines were significantly less affected in comparison, showing survival rates of 66% (HFF1) and 44% (HOB). On average, all cancer cell lines showed significantly lower surviving fractions (18.6%) than the normal human cell lines (56%) after 60 min exposure to Cypep-1.

### LIVE/DEAD Assay

**Figure 3:**
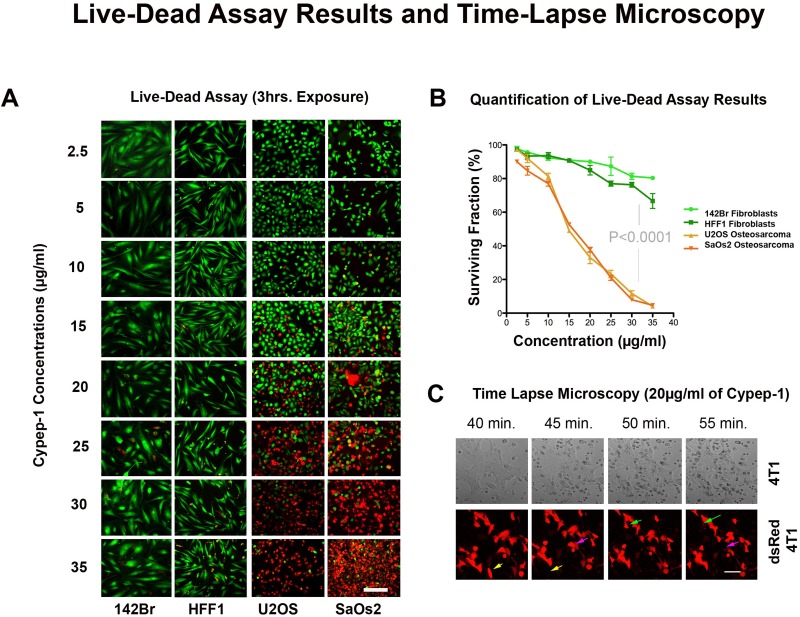
Effect of Cypep-1 on human osteosarcoma cell lines and normal human skin fibroblasts (142Br and HFF1) A) Representative images of cells treated with increasing concentrations for 3 hours. Green fluorescence (Calcein) indicates live cells and red (Ethidium homodimer-1) labels dead or dying cells. B) Dose-response curves based on the presence of Calcein. C) Time lapse microscopy of the mammary carcinoma cell line 4T1 treated with Cypep-1.

Visible cytotoxic effects on the osteosarcoma cell lines (U2OS, SaOs2) could already be observed after 3 hours incubation time with 10 µg Cypep-1/ml (Fig. 3A and B), which swiftly increased with higher peptide concentrations. Normal human fibroblasts (142Br, HFF1) were again significantly less affected (p<0.0001), displaying the first signs of cytotoxicity at an elevated concentration of 25 µg Cypep-1/ml. According to morphometric evaluation (Fig.3B), the average surviving fractions of the osteosarcoma cell lines were lower than 5% after exposure to a maximum concentration of 35 µg Cypep-1/ml, while the viability for the corresponding fibroblast cell lines 142Br and HFF1 was 81% and 64%, respectively.

### Time Lapse Confocal Microscopy

To monitor the kinetics of Cypep-1 *in vitro*, time-lapse confocal microscopy was performed on monolayers of 4T1 murine mammary carcinoma cells incubated with 20 µg Cypep-1/ml for 3 hours. As shown in Fig. 3C (upper panel), the induced membrane disruption caused an extensive loss of cell content into the medium, the strongest effect occurring between 40 and 55 min (Suppl. Film 1). To further visualize this effect, the experiment was repeated with stably transfected dsRed 4T1 cells, exposing them to the same peptide concentration and incubation period. A sequential, but immediate leakage of dsRed was observed between 40 and 45min, as well as between 50 and 55 min of exposure time to Cypep-1, indicating membrane destruction (arrows in Fig. 3C; Suppl. Film 2).

### Functional Interactions with Biological Model Membranes

Further insights on the interaction of Cypep-1 with biological model membranes were obtained by SPR as well as liposome-content leakage assays monitored by fluorescence spectroscopy.

### Surface Plasmon Resonance (SPR) Experiments

**Figure 4:**
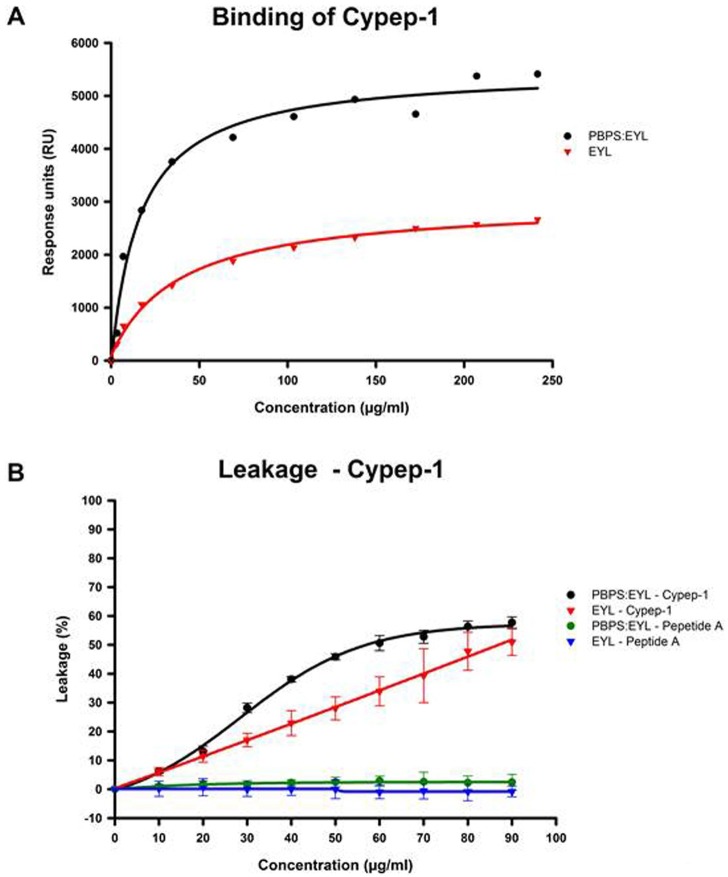
Quantification of Cypep-1s binding to negatively charged and neutral liposomes A) Surface plasmon resonance demonstrates a significantly higher affinity of Cypep-1 to negatively charged PBPS: EYL liposomes (blue) than to neutral EYL liposomes (green). B) Quantification of vesicle leakage of negatively charged PBPS: EYL (blue) and neutral EYL (green) liposomes in the presence of by Cypep-1 (circle) and the control peptide (triangle).

As shown in Fig. 4A, SPR revealed that Cypep-1 bound to both the negatively charged (PBPS: EYL) as well as neutral (EYL) liposomes. From the curve fittings of the data shown in Figure 4A, the peptide concentration of half-maximal binding (S_0.5_), a measure of the inverse of the apparent association or partition constant (1/Ka (app)), was calculated to provide a comparative and operational measurement for affinity. The resulting S_0.5_ values showed that Cypep-1 bound with significantly higher affinity to the negatively charged PBPS: EYL liposomes (S_0.5_ = 16.2 ± 4.4 µg/ml (4.7 ± 1.0 µM)) than to the neutral EYL liposomes (S_0.5_ = 39.1 ± 5.3 µg/ml (11.3 ± 1.5 µM)).

### Liposome Leakage Assays

The release of the fluorophore content from the respective liposomes in the presence of Cypep-1 and the control peptide (Peptide A) is depicted in Fig. 4B. A significant leakage of ANTS was observed for both types of liposomes in the presence of Cypep-1. At the intermediate concentrations tested (30-70 µg Cypep-1/ml dist. H_2_O), the negatively charged PBPS: EYL liposomes were clearly more affected by Cypep-1 than the neutral EYL liposomes. However, a similar extent of release was obtained for both liposome types at the maximum concentration (90 µg Cypep-1/ml). In both cases, the leakage upon exposure to Cypep-1 occurred in a dose dependent manner. Basically no change in fluorescence was observed in either of the liposome mixtures when exposed to the control peptide.

The S_0.5_ value provides an estimation of Cypep-1 concentration needed for half-maximal binding. No satisfactory estimation of S_0.5_ could be obtained for the disruption of neutral EYL liposomes by Cypep 1 due to lack of saturation at attainable peptide concentrations. Nevertheless the measurements clearly indicated a higher affinity of Cypep-1 towards the negatively charged PBPS: EYL liposomes than towards the neutral ones.

Comparing the binding (Fig. 4A) and disruption measurements (Fig. 4B), the concentration of Cypep-1 necessary to achieve membrane disruption in negatively charged vesicles (Fig. 4B; S_0.5_ = 8.3 ± 0.5 µM) was about two fold higher than the concentration required for half-maximal binding (Fig. 4A; S_0.5_ = 4.7 ± 1.0 µM), indicating that the peptide has to accumulate at the surface for disruption.

### Plasma Activity, Bio Distribution and *In Vivo* Toxicity of Cypep-1

#### Radio Labelling of Cypep-1

The elution pattern of ^125^I-labelled Cypep-1 corresponded to the small size and relative hydrophilicity of the peptide (27 amino acids, MW 3492.16), resembling closely that of unlabelled Cypep-1 as detected at UV 210 nm (data not shown).

#### Plasma Activity and Bio Distribution

**Figure 5:**
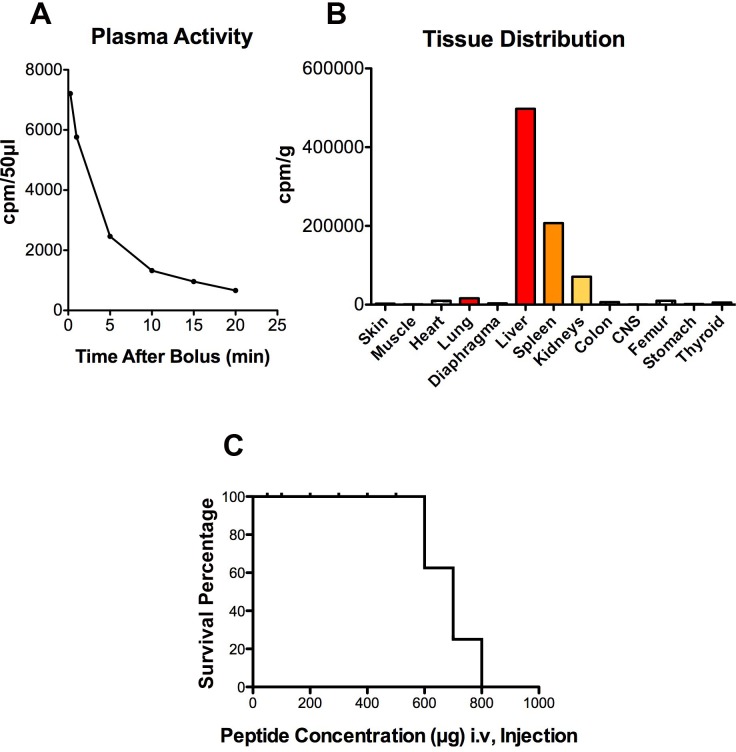
Pharmakokinetics, tissue distribution and toxicity after intravenous injection of ^125^I-labelled Cypep-1 A) Plasma clearance after bolus injection of 50 µg of Cypep-1. B) Tissue Distribution of Cypep-1 20 min after administration C) *In Vivo* toxicity.

The plasma activity of ^125^I-Cypep-1 declined rapidly over the course of 20 min, the most dramatic drop occurring within the first 5 min following the i.v. tracer injection. Correspondingly, the plasma half-life of Cypep-1 was approx. 3.15 min (Fig. 5A).

As depicted in Fig. 5B the tissue distribution of Cypep-1 showed outstandingly high concentrations (* plasma space per ml / g organ tissue wet weight) in liver (11.74*), spleen (4.89*) and kidneys (1.685* per kidney). Comparatively low concentrations were found in the lungs (0.38*), stomach (0.04*), jejunum (0.17*), colon (0.14*) and muscle tissue (0.02*). Similarly, a low bio distribution value was obtained for the thyroid gland (0.13*). Very low concentrations of Cypep-1 were measured within the organs of the central nervous system, e.g. cerebellum (0.00*), cerebrum (0.01*) and eye (0.02*).

#### In *Vivo* Toxicity

Cypep-1 was slowly administered into the tail vein of 37 BALB/c mice at increasing doses from 50 to 800 µg Cypep-1 per 100µl bolus per animal (Fig. 5C). At Cypep-1 concentrations up to 500 µg, no signs of distress or pain were found in the experimental animals. The first signs of toxicity were observed at concentrations of 600 µg of Cypep-1, whereby the affected animals fell into a seizure-like catatonic stupor immediately after peptide administration. The first 5-10 min post Cypep-1 injection then proved to be decisive: Some experimental animals recovered within this period of time (after doses up to 700 µg Cypep-1) and then continued to behave normally, while those that died, first began to show signs of laboured breathing, which then exacerbated and finally lead to death within the same period of time (5 - 10 min after the i.v. injection (maximum)).

The detailed autopsies following all cases of lethal toxicity revealed no macroscopic changes or anomalies. Thus, neither hints as to a possible mechanism of systemic toxicity of Cypep-1 for doses higher than 600 µg (24 mg/kg body weight), nor evidence as to the actual cause of death upon exposure to lethal doses of 800 µg (32 mg/kg body weight) could be obtained. Other than the seizure-like catatonic stupor described above, the experimental animals showed no further signs of distress, pain or potential toxic effects after administration of Cypep-1. During the continuous observation period of the first 2 hours post injection, the mice (even the ones that had presented with severe signs of catatonic stupor before, but had recovered) continued their habitual behaviour, without any signs of discomfort, stress or pain. Further check-ups at 4 h, 6 h, 12 h and 24 h post Cypep-1 injection again showed mice at normal activity levels in accordance with their day/ night rhythm.

#### Treatment of 4T1 Murine Breast Cancer Allografts

**Figure 6:**
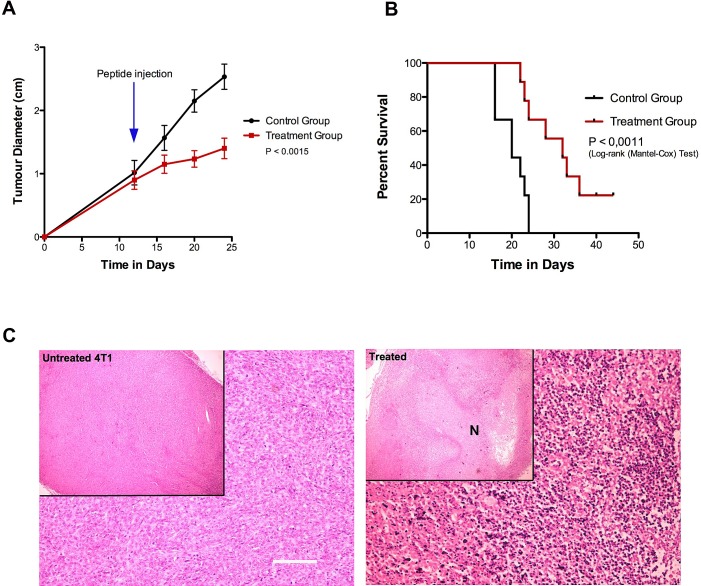
Treatment of 4T1 murine breast cancer allografts with Cypep-1 A) A single intratumoral injection of 1200 µg Cypep-1 significantly reduced tumour growth (P<0.0001) and B) increased survival in the treated animals compared to the control group (P<0.0011). C) Representative histology from untreated (left) and treated (right) tumors showed extensive areas of necrosis in the treatment group.

As shown in Fig. 6A, all experimental animals were subjected to intratumoural injections at day 12 post-tumour inoculation, when the tumours of both study groups had reached an average volume of 0.12cm^3^. The animals of the treatment group received a single intra-tumoural injection of 1200 µg of Cypep-1 dissolved in 300µl 0.9% NaCl, while the animals of the control group were administered a similar injection volume of 0.9% NaCl. None of the animals showed any signs of discomfort, pain or toxicity after the respective injections. Again, once recovered from the Isofluoran anaesthesia, they all continued their habitual behaviour at normal activity levels.

At day 24, a significant reduction in tumour growth was observed in the tumours of the treatment group with an average volume of 1.1 cm^3^, whereas the control tumours had grown to an average size of 3.5 cm^3^ (P<0.0001). The survival analyses (Fig. 6B) showed that the animals assigned to the treatment group lived significantly longer (median survival 32 days) than those of the control group (median survival 20 days) (P<0.0011).

Histological analysis of the untreated control tumours revealed a uniform tumour mass with no evidence of necrosis. The tumour cells had an atypical cell appearance and were densely packed with numerous mitotic figures (Fig 6C; left panel). In contrast, the tumours in the treatment group had extensive areas of necrosis at 24 hours after intra-tumoural Cypep-1 administration. Especially the border zone between viable and necrotic tissue contained large areas of pyknotic cells, characterized by an irreversible condensation of chromatin in the nucleus (Fig 6C; right panel).

### DISCUSSION

In this study, we designed a proteolytically stable cationic lytic peptide of 27 D-amino-acids, designated Cypep-1, which showed a strong cytotoxic effect on a large panel of tumour cell lines, while affecting normal human fibroblasts to a significantly lesser extent.

SEM micrographs revealed numerous holes in the plasma membrane of 4T1 mammary carcinoma cells already after 60 min exposure to 35 µg/ml Cypep-1, while a subsequent total disintegration of the cell membrane occurred after 6 hours (Fig. 1C-F). This hole forming capacity has been described earlier for the AMPs Magainin and Dermaseptin S4, where it was named “toroidal pore” or “wormhole model” [52-56]. It has been viewed as a possible early step prior to the collapse of the membrane in the context of the “carpet model” mechanism, a common mechanism of action of many AMPs, whereby they assemble on the outer membrane surface of their target, forming ”a carpet”, until their local concentration is sufficient to increase the curvature strain on the target membrane to the point of formation of local “toroidal pores” [12, 55, 57]. These membrane perforations are thought to facilitate the passage of low molecular weight molecules prior to complete membrane lysis [16, 30]. The formation of microvilli and filopodia, as seen in the untreated U87 cells (Fig. 1.A-B) may also add to the susceptibility towards Cypep-1 [47].

The membranolytic effect of Cypep-1 was confirmed by time-lapse confocal microscopy, where exposure of 4T1 and dsRed4T1 murine mammary carcinoma cells to Cypep-1 resulted in extensive membrane leakage (Fig. 3C; Suppl. Films 1 and 2).

SPR experiments (Fig. 4A), as well as liposome leakage assays (Fig. 4B) on negatively charged and neutral liposomes [30, 58], showed that Cypep-1 bound to both types of liposomes. Although its affinity to negatively charged vesicles was significantly higher, these results were slightly unexpected in view of Cypep-1's high isoelectric point (pI=11.81) and the generally low affinity of AMPs towards the zwitterionic neutral membranes of normal cells. However, similar observations have been reported. Both Dermaseptin S4 [59] and human-like Cecropin LL-37 [60] were found to have a strong binding affinity and permeation capability for both zwitterionic and negatively charged phospholipid vesicles. Likewise was their relatively high affinity towards zwitterionic PC phospholipid membranes deemed surprising given their high positive net charge. This was explained in both cases by a possible interaction of the respective hydrophobic N-termini of the peptides with the hydrophobic parts of the neutral zwitterionic phospholipids on the membrane surface of their targets, suggesting that a bundling of such interactions could initiate the binding of the respective peptides also to membrane of neutral net charge [30].

In sum, these findings are in line with the “carpet model” for membrane disruption, which was first proposed to describe the mode of action of Dermaseptin S4 [58] and later on that of many other AMPs [56, 59, 61-67].

The dose dependent cytotoxic effect of Cypep-1 was demonstrated *in vitro* for the concentration range of 2.5to 35 µg Cypep-1/ml, using two different cell viability assays. However, our results obtained using the LIVE/DEAD assay (Fig. 3A and B) indicated a much wider therapeutic window, showing considerably higher cell survival rates for the human fibroblasts (142Br (human skin) and HFF1 (human foreskin)) and even lower survival rates in the osteosarcoma cell lines (U2OS and SaOs2) than the corresponding MTT results (Fig. 2A and B; Suppl. Fig. 2). Both cell assays measure the metabolism in intact cells. In case of the MTT proliferation assay, the applied Thiazolyl Blue Tetrazolium Bromide is metabolized only by viable cells, resulting in the formation of MTT-Formazan crystals, which are then solubilised in acidified isopropanol and immediately measured at 570 nm by means of a microplate reader.

The LIVE/DEAD assay contains Calcein-AM and Ethidium-Homodimer-1 and thus stains both live and dead cells simultaneously. Viable cells are able to convert non-fluorescent Calcein-AM into green fluorescent Calcein enzymatically due to the ubiquituous presence of intracellular esterase activity, whereas the red fluorescent Ethidium-Homodimer-1 is excluded by their intact cell membranes. Thus, the penetration of Ethidium-Homodimer-1 indicates a loss of plasma membrane integrity, staining dead and dying cells. We therefore conclude that, in view of their membranolytic mode of action, the LIVE/DEAD assay may provide a more accurate and sensitive method for the evaluation of cell death induced by cytolytic peptides.

The biodistribution of ^125^I-labelled Cypep-1 showed a rapid initial plasma clearance, as may be expected due to the fast distribution within the extracellular space of a small 27 amino acids molecule (Fig. 5A). Its subsequent slower plasma clearance reflected both its removal due to glomerular filtration and tubular degradation [68], as well as its high uptake into the reticuloendothelial system (Fig. 5B). Correspondingly, Cypep-1 was found to have a short plasma half-life (approx. 3.5 min). In contrast, hardly any accumulation was found in the lungs, organs of the gastro-intestinal tract, muscle tissue (Fig. 5B). This suggests that Cypep-1 is probably not suited for systemic administration in its present form. Still, one might try to exploit the pronounced retention of Cypep-1 in the liver and spleen to possibly achieve an oncolytic effect against localized cancers in these organs.

The low biodistribution value for the thyroid gland (0.13*) reflected the absence of free ^125^I-labelled iodine, thus being in line with the reverse phase chromatography results. Very low concentrations found within the organs of the central nervous system indicated that Cypep-1 did not pass the blood-brain-barrier (Fig. 5B).

Detailed autopsies were unable to disclose a possible mechanism of systemic toxicity, such as haemorrhaging or organ failure. Likewise, the reason for the seizures due to toxic doses remains unknown. In view of the strong evidence that Cypep-1 did not pass the blood-brain-barrier, it seems highly unlikely that these seizures could have been provoked by the penetration of Cypep-1 into the central nervous system. However, given its cationic nature, the quick onset of signs of toxicity and thus provoked changes in the breathing pattern of the affected animals may possibly point towards a shift in the electrolyte pattern. This should clearly be explored further.

Achieving suitability for overall systemic use of Cypep-1 in the future would probably entail modifications of the present peptide. However, inspiration might be drawn from peptide studies of other groups, e.g. the creation of chimeric peptides, linkage to known receptor-ligands [69], hormone ligands [70, 71] or other nano carriers, as previously done with Melittin [72, 73].

To assess the effect of Cypep-1 on solid tumours *in vivo*, we injected the peptide locally into 4T1 murine mammary carcinoma allografts. A significant inhibition of tumour growth was achieved (Fig. 6A), resulting in large areas of severe necrosis (Fig. 6C) within the treated tumours (Fig. 6C). This increased the survival of the treated animals (Fig. 6B) significantly and there were no signs of systemic toxicity. This suggests that Cypep-1 in its present form appears to be well suited for local treatment of solid tumours.

In conclusion, we have designed a novel cytolytic peptide that shows a strong cytotoxic effect *in vitro* against various cancer cell lines. Functional studies using both cell lines and artificial model membranes, indicate that its effect is caused primarily by its interaction with negatively charged plasma membranes. Evidence suggests that Cypep-1 first accumulates on the membrane of its target, where it induces the formation of holes within the first 30 min upon application, while ultimately leading to its total disintegration within 6 hours. These findings support the “carpet model” of membrane disruption as its mechanism of action. However, it still remains to be investigated whether Cypep-1 may possible have any pro-apoptotic and/or anti-angiogenic effects.

Cypep-1's relative selectivity towards cancer cells, apparent proteolytic stability and low systemic toxicity suggest a considerable therapeutic potential.

Given its substantial local anti-tumoural effects on solid tumours *in vivo*, we believe that Cypep-1 should be exploited further for local administration against solid tumours.

## MATERIALS AND METHODS

### Peptide Design and Production

The initial peptide library was designed *in silico* with respect to the identification of active sites of tumours suppressors and oncogenes by combining bio-informatic softwares provided by *Expasy* and the data base *UniProtKB/ Swiss-Prot*, which are both available online [51, 74]. The sizes of the resulting *in silico* peptides varied from 10 to 30 amino acids. In order to facilitate their cellular internalisation, a TAT-HIV sequence was attached to the C-terminal ends. All peptides were C-terminal acetylated and N-terminal amidated. The peptide library was produced by Cambridge Peptides (Cambridge, UK). The peptides were then screened with the MTT (3-(4,5-dimethylthiazol-2-yl)-2,5-diphenyltetrazolium bromide) assay on U87 human glioma cells. Cellular morphology after peptide exposure was assessed by crystal violet staining and light microscopy. All experiments were performed in triplicate.

The peptide with the most potent activity was then selected for further analysis. To increase the stability of the peptide, two further sub-formulations of the original formula (L-aa, containing only L-amino acids) were created: D-aa (retro inversed, (ri)), a peptide comprised of a backward sequence of the original *in silico* formula, yet made up solely of D-amino acids and an analogue peptide to original *in silico* peptide formula, comprised of D-amino acids. D-amino acids are highly resistant to protease mediated degradation and have a low immunogenic response. The respective peptide variants were tested in the following concentrations: 7.5, 10, 15, 20 and 25 µg/ml and incubated for 3 hours. The activity (at a concentration of 15 µg/ml) of the most potent of these three peptide variants was verified again after an incubation time of 3 hours by means of light microscopy. This novel cationic peptide of 27 amino acids (YGRKKRRQRRRGKTLRVAKAIYKRYIE) was then selected for larger scale synthesis, performed by GenScript (Piscataway, NJ, USA).

### Scanning Electron Microscopy (SEM)

4T1 mammary carcinoma cells growing on cover-glass slides were exposed to 35 µg/ml Cypep-1 for 60 minutes and for 6 hours, respectively. Thereafter, the slides were prepared for SEM as originally described by Anderson [75]. After mounting them on stubs with tape and silver paint, an approximately 50 nm thick gold layer was evaporated on the specimens, using a Polaron E5000 SEM Coating Unit (Polaron Components Ltd, Watford, UK). Finally, the specimens were examined with a Jeol JSM-7400F scanning electron microscope (Jeol Ltd, Tokyo, Japan).

### Cell Lines and Cell Culture

Cypep-1 was tested in various concentrations on a total of 17 cell lines, which included 13 human and 1 murine tumour cell line(s), as well as 3 normal human cell lines. Listed in detail: 4 human osteosarcoma (U2OS, SaOS2, HOS and MNNG-HOS), 6 human glioblastoma (A172, GaMg [76], LN18, U87, U373 and T98G) and 3 human mammary carcinoma cell lines (MCF7, AU565 and MDA-MB-468) as well as an additional murine mammary carcinoma cell line (4T1). Furthermore, 3 normal human cell lines were included: 2 normal human fibroblast cell lines (HFF1 and 142Br) as well as normal human osteoblasts (HOB).

The tumour cell lines were obtained from the American Type Culture Collection (ATCC, Rockville, MA), with the exception of GaMg, which had originally been established at the University of Bergen (Department of Biomedicine, Bergen, Norway) [76].

All cell lines used were DNA fingerprinted by short tandem repeat (STR) analysis.

Most cell lines were grown in Dulbecco's Modified Eagle's Medium (DMEM; Sigma-Aldrich, St. Louis, MO), supplemented with 10% foetal calf serum (FCS; PAA Laboratories GmbH, Pasching, Austria), 2% L-glutamine (Cambrex, East Rutherford, NJ), four times the prescribed concentration of non-essential amino acids (NEAA 100x; Cambrex, East Rutherford, NJ), and the antibiotics Penicillin (100 U/ml) and Streptomycin (100 mg/ml (PEN-STREP™; 5000U Penicillin/ml and 5000µg Streptomycin/ml; Cambrex, East Rutherford, NJ), as well as Plasmocin™ (25mg/ml; Invivogen, San Diego, CA).

All normal human cell lines were purchased from Promo Cell (Heidelberg, Germany). While the normal human foreskin fibroblasts (HFF1) were grown in DMEM medium as described above, the additional human skin fibroblast cell line (142Br) and the normal human osteoblasts (HOB) were cultured in purchased cell type specific growth mediums (Promo Cell, Heidelberg, Germany).

All cell lines were kept in a standard tissue culture incubator at 37°C and 5%CO_2_ in 75cm^2^ tissue culture flasks (Nunc™, Roskilde, Denmark). They were passaged at approx. 80% confluence and allowed a settling period of 24 hours before the start of the respective experiments.

### Cell Viability Assays

The *in vitro* cytotoxicity of Cypep-1 was measured by the MTT proliferation assays and a LIVE/DEAD Viability/ Cytotoxicity assay kit (Molecular Probes, Invitrogen, Invitrogen Dynal AS, Oslo, Norway).

### MTT Proliferation Assays

MTT proliferation assays were performed on all cell lines (with the exception of 142Br (human skin fibroblasts)), using monolayers of 15 000 cells in sterile 96 well tissue culture plates (Nunc™, Roskilde, Denmark), respectively. All cells were incubated with Cypep-1 dissolved in 0.9% NaCl, at the following concentrations: 0.1, 0.5, 1.0, 5, 10, 15, 20, 25, 30 and 35µg of Cypep-1 per ml and assayed after 15, 30, 45, 60, 75, 90, 180, 270 and 360 min.

### LIVE/DEAD Viability/Cytotoxicity Assays

In order to visualise and quantify the cytotoxic action of Cypep-1 simultaneously, the LIVE/DEAD® Viability/Cytotoxicity Kit was employed according to the manufacturers instructions on monolayers of 30 000 cells in sterile 24 multi well dishes (Nunc™, Roskilde, Denmark), using the selected human osteosarcoma cell lines U2OS and SaOs2 as well as the two human fibroblast cell lines HFF1 (foreskin) and 142Br (skin).

The monolayer cultures were incubated with Cypep-1 for a period of 3 hours in concentrations ranging from 2.5 µg to 35 µg/ml. The cells were observed using an inverted fluorescence microscope (Nikon Eclipse 2000E, Tokyo, Japan) equipped with FITC (green fluorescence) and TRITC (red fluorescence) filter optics. The fluorescent cells were then photographed at a 10x magnification. The proportions of viable cells (live cells showing green fluorescence due to the presence of Calcein) and dead cells (red fluorescent due to the membrane penetration of Ethidium-Homodimer-1) were quantified using a Lucia G Morphometry System, Version 4.71 (Laboratory Imaging, Prague, Czech Republic).

### Time Lapse Confocal Microscopy

30.000 cells of the murine mammary carcinoma cell line 4T1 were seeded into a sterile 24 well dish (Nunc™, Roskilde, Denmark). After 24 hours, the monolayer culture was treated with 20µg Cypep-1/ml, followed by immediate observation of the cells at 37°C and 5% CO_2_ for a period of 3 hours by means of time-lapse confocal microscopy, using a Zeiss LSM510 Meta System (Carl Zeiss AG, Jena, Germany), equipped with both bright field optics and red fluorescence optics at 595nm. The same experiment was repeated as described above with 4T1 cells that had previously been stably transfected with red fluorescent protein, using a dsRed-expressing lentiviral vector pWPXL-dsRED as previously described by Niclou et al. [77].

### Interactions of Cypep-1 with Biological Model Membranes

Structural and mechanistic insights on the interaction of Cypep-1 with biological model membranes were obtained by surface plasmon resonance (SPR) and liposome-content leakage assays monitored by fluorescence spectroscopy.

### Surface Plasmon Resonance (SPR) Experiments

SPR was used to quantify the binding of Cypep-1 to phospholipid model membranes of different compositions at neutral pH (pH 7.4) and a temperature of 25 °C. 100 nm liposomes made of egg yolk lysolecithin (EYL; L-α-phosphatidylcholine; 95% purity) were employed to represent amphitropic, neutral membranes, while liposomes composed of both pig brain L-α-phosphatidylserine (PBPS) and EYL (from Avanti Polar Lipids Inc., Alabaster, Alabama, USA), with PBPS:EYL in a 1:1 M: M mixture, represented acidic, negatively charged membranes. Both liposome type mixtures have been found to properly represent the fluidity of biologically relevant membranes [78] [79]. The SPR analyses were carried out on a BIAcore 3000 instrument (BIAcore AB, Uppsala, Sweden) as described in Halskau et al. [80], using a L1 sensor chip from BIAcore (BIAcore AB, Uppsala, Sweden).

### Liposome Leakage Assays

To assess the membranolytic effect of Cypep-1 on the integrity of phospholipid model membranes, liposome leakage assays monitored by fluorescence spectroscopy were performed on 200 nm liposomes as described earlier [81, 82]. A 18-residue peptide that shows a low interaction with membranes[82] (purchased from CPC Scientific, San Jose, CA) was used as control in the leakage assays (Peptide A).

Negatively charged PBPS: EYL (1:1 mixture) as well as neutral pure EYL vesicles were used, in which both liposome types encapsulated the fluorochrome ANTS (8 aminonaphtalene-1,3,6 trisulfonic acid) and its corresponding quencher DPX (p-xylene-bis[N-pyridinium bromide]). In principle, a disruption of the liposomes' bi-layer induces leakage of the fluorophore ANTS. This entails an increase in fluorescence at 510 nm. All fluorescence spectra were recorded at 25°C and pH 7.4 on a Cary Eclipse Fluorescence Spectrophotometer (Varian Optical Spectroscopy Instruments, Mulgrave, Victoria, Australia). The respective titrations with Cypep-1 and the control Peptide A were performed in concentrations ranging from 0 µg/ml to 90 µg/ ml (0 µM to 26 µM). The fluorescence was measured for each concentration point of the concentration series, and all measurements were performed in triplicate. At the end of each measurement, Triton X-100 was added to a final concentration of 2 mM, to obtain the maximum membrane leakage. The resulting maximum fluorescence at 510 nm is defined as 100%, i.e. complete liposome breakdown. The induced leakages of the fluorophore ANTS due to exposure to the peptides were then evaluated as a percentage of its complete release after Triton X-100 exposure. The initial fluorescence intensity of the respective liposome solutions was defined as 0% leakage.

### Plasma Activity, Bio Distribution and *In Vivo* Toxicity of Cypep-1

### Radio Labelling of Cypep-1

The radio labelling of Cypep-1 was performed with ^125^I, according to the Iodo-Gen method as described by Fraker et al. [83]. The obtained ^125^I-Cypep-1 stock solution was stored in the dark at 4°C for 48 hours prior to validation by reversed phase chromatography, using a 4.6 × 50 mm Dionex Proswift RP-2H column on an Ettan LC System (GE Healthcare, Oslo, Norway) in line with a Radiomatic 610TR Flow System Analyzer by Perkin Elmer (PerkinElmer, Waltham, MA) for gamma detection.

### Plasma Activity and Biodistribution of Cypep-1

The plasma activity and biodistribution of ^125^I-Cypep-1 was evaluated in a single experiment. An adult female Wistar rat of 260 g body weight was anaesthetized using 15 mg of Pentobarbital i.p.. Its body temperature was maintained at 37°C, using both a heating pad and an infrared lamp. Two PE-50 polyethylene catheters were placed into the femoral vein for the bolus injection of Cypep-1 and one into the femoral artery for blood sampling. 50 µl of the ^125^I-Cypep-1 stock solution dissolved in 500 µl 0.9% NaCl were injected i.v., and a total of six arterial blood samples (100 µl per sample) were collected at 0.25, 1, 5, 10, 15 and 20 min following the ^125^I-Cypep-1 administration. The animal was sacrificed under deep anaesthesia with an i.v. bolus injection of 0.5 ml saturated KCl. The tissue and plasma samples were harvested immediately and counted for ^125^I-activity in a gamma-counter (LKB Wallac 1285, Turku, Finland). The peptide distribution volumes in the various body tissues were calculated as the ^125^I-activity per gram tissue sample divided by the time-averaged plasma ^125^I-activity during the 20 minutes clearance period (*= plasma equivalent space in ml/g tissue wet weight).

### *In Vivo* Toxicity of Cypep-1

Cypep-1 was injected into the lateral tail vein of 37 BALB/c mice (average age 131,7 days; average weight 25.2 g). In detail, the group consisted of 18 male (average weight 26.5g) and 19 female animals (average weight 23.9g). The respective peptide concentrations administered were increased systematically from 50µg up to 800µg Cypep-1 per bolus per animal. All Cypep-1 doses were adjusted to a total injection volume of 100µl 0,9% NaCl, which remained constant for all animals. Throughout the experiment, a group of 4 animals (2 males/ 2 females) received the same Cypep-1 concentrations per bolus, respectively. All injections were performed very slowly in order to avoid cardiac stress. The mice were then observed for a period of 24h with regards to signs of distress and pain (e.g. orbital tightening, flattened ear position, formation of nose and/or cheek bulge, whisker change, overall activity level) as well as potential toxic effects. During the first 2 hours, the animals were monitored continuously and then checked again at 4 h, 6 h, 12 h and 24 h post injection.

### Treatment of Subcutaneous 4T1 Metastatic Breast Cancer Allografts

10^6^ 4T1 murine metastatic breast cancer cells were grafted subcutaneously under Isofluran anaesthesia into the right upper quadrant of the back of 20 female BALB/c mice (average age 126,25 days; average weight 23.8g). At day 12, when the tumours of both groups had reached a tumour volume of 0.12cm^3^ in average, 10 female mice (average weight 23.9 g) received a single intra-tumoural injection of 1200 µg of Cypep-1 dissolved in 300 µl 0.9% NaCl, whereas an equal injection volume of 0.9% NaCl was administered to the 10 animals of the control group (average weight 23.7 g). All injections were performed under isofluoran gas anaesthesia. The animals were then observed for signs of distress as described above. 24 hours after the injections, two animals (one assigned to the control and one of the treatment group) were sacrificed for histological analysis. The harvested tumours were immediately fixed in 4% buffered formaldehyde and embedded in paraffin, cut into 5 µm sections and stained with H&E.

In the remaining 18 experimental animals, the tumour size was measured at regular intervals (every 4^th^ day) until day 24 post tumour grafting. All mice were monitored daily for signs of distress and pain. Those that became sick were euthanized humanely. All animal procedures described were in accordance with protocols approved by The National Animal Research Authority (Oslo, Norway).

### Statistical analyses

Statistical analyses were performed using Prism software. Comparisons between groups were performed using one-way analysis of variance (ANOVA). For the analysis of the Kaplan-Meier a log-rank (Mantel-Cox) test was used. P<0.05 was considered significant.

## SUPPLEMENTARY FIGURES AND MOVIES






