# Supplementary material for: Identification of a novel lytic peptide for the treatment of solid tumours
Source: Genes Cancer. 2014 May;5(5-6):186–200. doi: 10.18632/genesandcancer.18 (PMC4104761; doi:10.18632/genesandcancer.18)
Supplement: Supplementary file 1 [file ganc-05-186-s001.pdf]

Identification of a novel lytic peptide for the treatment of solid tumours

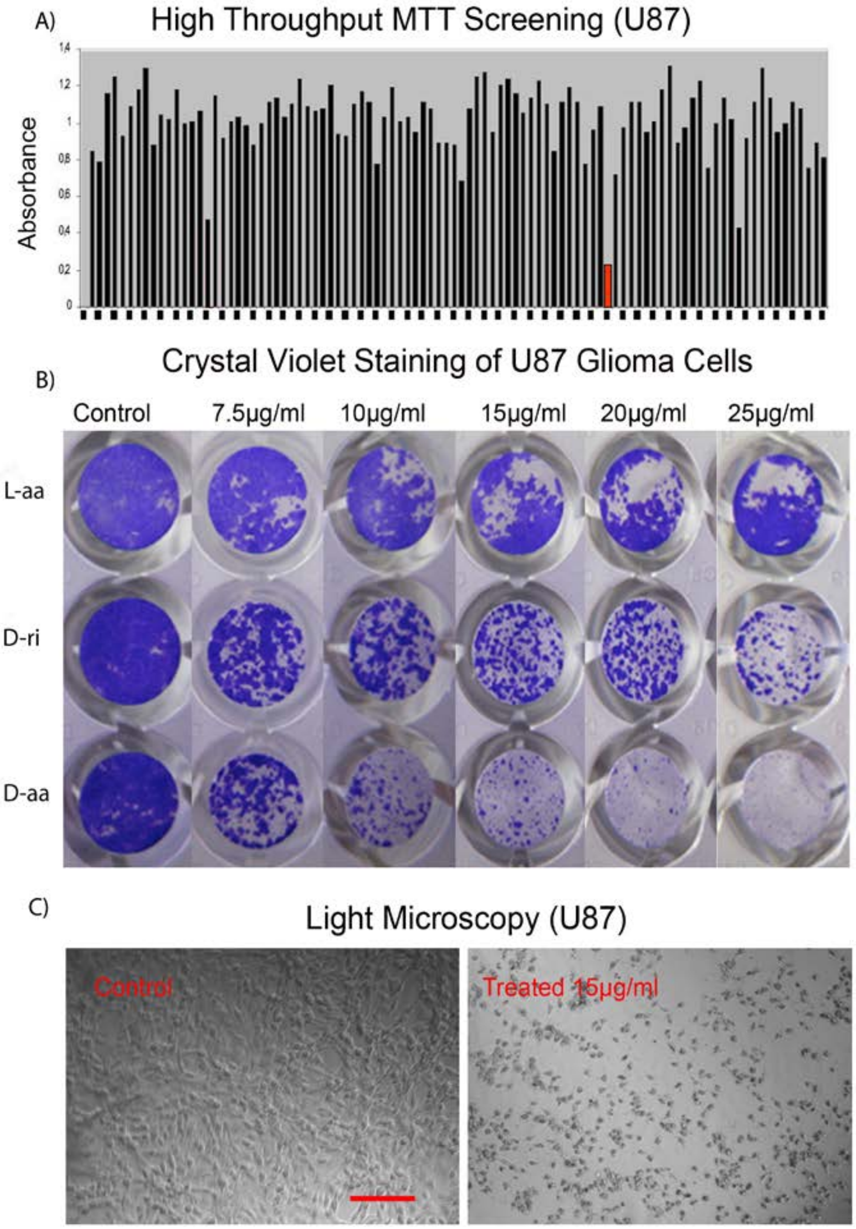

Supplementary figure 1: Initial screening of peptide library and identification of cancer cell cytotoxic peptides. A) Screening of 96 peptides identified 3 peptides with potent cytotoxic effect, Cypep-1 being the most effective (red bar). B) Substituting L- with D-amino acids increases stability and potency of Cypep-1. C) Light microscopy showing U87 monolayers before (left) and after (right) treatment with Cypep-1.
